# A Multilayer Nonlinear Permutation Framework and Its Demonstration in Lightweight Image Encryption

**DOI:** 10.3390/e26100885

**Published:** 2024-10-21

**Authors:** Cemile İnce, Kenan İnce, Davut Hanbay

**Affiliations:** 1 Department of Computer Engineering, İnönü University, Malatya 44280, Türkiye; cemile.ince@inonu.edu.tr (C.İ.); davut.hanbay@inonu.edu.tr (D.H.); 2 Department of Software Engineering, İnönü University, Malatya 44280, Türkiye

**Keywords:** chaotic permutation, cryptography, image encryption, lightweight encryption, nonlinear permutation

## Abstract

As information systems become more widespread, data security becomes increasingly important. While traditional encryption methods provide effective protection against unauthorized access, they often struggle with multimedia data like images and videos. This necessitates specialized image encryption approaches. With the rise of mobile and Internet of Things (IoT) devices, lightweight image encryption algorithms are crucial for resource-constrained environments. These algorithms have applications in various domains, including medical imaging and surveillance systems. However, the biggest challenge of lightweight algorithms is balancing strong security with limited hardware resources. This work introduces a novel nonlinear matrix permutation approach applicable to both confusion and diffusion phases in lightweight image encryption. The proposed method utilizes three different chaotic maps in harmony, namely a 2D Zaslavsky map, 1D Chebyshev map, and 1D logistic map, to generate number sequences for permutation and diffusion. Evaluation using various metrics confirms the method’s efficiency and its potential as a robust encryption framework. The proposed scheme was tested with 14 color images in the SIPI dataset. This approach achieves high performance by processing each image in just one iteration. The developed scheme offers a significant advantage over its alternatives, with an average NPCR of 99.6122, UACI of 33.4690, and information entropy of 7.9993 for 14 test images, with an average correlation value as low as 0.0006 and a vast key space of $2^{800}$. The evaluation results demonstrated that the proposed approach is a viable and effective alternative for lightweight image encryption.

## 1. Introduction

Cryptography is a critical tool for protecting digital data from unauthorized access in our increasingly digital world [1]. It is the practice of securing information by transforming it into an unreadable format, called ciphertext, using mathematical algorithms. This process prevents unauthorized individuals from accessing the information’s original content. The transformations used in cryptography are not always “reversible”, but it is in the encryption. While some encryption algorithms involve decryption keys which allow authorized users to retrieve the original data, others are designed to be irreversible, permanently obscuring the information [2]. Multimedia data such as images and videos often require specialized techniques due to their unique characteristics, yet traditional encryption algorithms can be used. The strong spatial and tonal correlation between nearby pixels in digital images leads to significant redundancy [3,4].

Image encryption, similar to other cryptographic fields, relies on two core processes: confusion and diffusion. During the confusion stage, pixel positions are rearranged, effectively scrambling the image content. The subsequent diffusion stage incorporates both the secret key and the original image through complex mathematical functions. This integration ensures that even minor changes in image data do not significantly impact overall security. However, the high computational complexity of encryption methods makes them unsuitable for resource-constrained devices like the Internet of Things (IoT) [5,6], whose use has exploded across various industries and daily life. Therefore, lightweight encryption algorithms and naturally lightweight image encryption algorithms have been developed to address these limitations, aiming to balance security with efficiency in resource-constrained devices.

The primary objective of lightweight image encryption is to shield images from unauthorized access while adhering to strict limitations on processing power and memory consumption. This process typically unfolds in two distinct stages: confusion and diffusion. During the confusion phase, pixels undergo random positional alterations. Subsequently, the diffusion phase entails modifying the individual pixel values. To align with the intended development approach, all operators employed in both the confusion and diffusion stages must be meticulously selected from computationally efficient methods [7].

Various methods are available for the pixel permutation step. These methods include row or column circular shifting [8,9], 2D matrix transformation, zigzag [10], spiral [11,12], and cornering [13] algorithms. They can be employed during different stages of image encryption, such as the confusion step, the diffusion step, or even both. Furthermore, while these methods can be used independently for permutation, there are also hybrid algorithms which combine these approaches [7,14]. However, an ideal encryption scheme should not try to be a one-size-fits-all solution for every confusion or diffusion algorithm. At its most basic, the proposed algorithm should have a flow of steps based on analytical reasons. All steps must be planned in detail, elegant, free from unnecessary complexity, and developed in accordance with the targeted system and working environment.

Another critical issue to consider when developing a lightweight algorithm is its resistance to post-quantum attacks. Research in this area suggests that increasing the key space size of existing algorithms could potentially create algorithms resistant to these attacks [15].

One of the most widely used permutation techniques is 2D matrix permutation [16]. This method utilizes two primary approaches. The first approach, as shown in Equation (1), employs a permutation matrix of the same size as the image, like S-boxes in block ciphers. The second approach, presented in Equation (2), utilizes two independent random arrays—one in the image height dimension and other in the image width dimension—to permute the image pixels:

(1) A[i,j]=I[x[i,j],y[i,j]]

(2) A[i,j]=I[x[i],y[j]]

In these equations, *I* represents the original image, and *A* represents the scrambled image. Both *x* and *y* are random matrices with dimensions M × N for Equation (1) and *M* and *N* for Equation (2), matching the size of the original image. These matrices are randomly generated using an ant pseudo-random number generator [17]. It is worth noting that the scheme’s security heavily relies on the chosen random number generation method [18,19]. Typically, chaotic equations are utilized for this purpose [20]. However, no matter how strong the permutation process is, permutation-based encryption alone is vulnerable to many attacks [21]. To address this, the permutation step can be either enhanced in complexity or combined with a propagation step. This is the approach adopted in the proposed method.

As suggested in [22], both 2D mapping methodologies are linear in one side of the permutation. Therefore, this study proposes nonlinear, two-stage, random sequence-based mapping in order to overcome this linearity. While one study [22] focused on specialized medical images, it does not directly translate to color images with RGB channels due to the inherent differences. Furthermore, existing research primarily concentrates on employing various chaotic maps and lacks a comprehensive encryption framework. As such, the scope of this work remains confined to specific domains. To address this limitation, this study proposes a novel approach which effectively handles three-channel color images while maintaining its effectiveness in medical images.

This work proposes an elegant, variable-step, chaos-based nonlinear 2D mapping framework utilized in color image encryption. The proposed method boasts five key advantages:

1. It introduces a flexible, chaos-based nonlinear mapping scheme with a variable number of iterations for color image encryption, allowing for tailored security levels.
2. The approach offers versatility. It can be used for permutation only or additional diffusion included during internal steps for enhanced security.
3. The design prioritizes efficiency, making it particularly well suited for lightweight image encryption algorithms.
4. This work introduces a novel and efficient permutation and diffusion scheme which addresses the limitations of relying solely on permutation steps in image encryption. This elegant design strikes a balance between security and efficiency, making it suitable for various applications.
5. To increase the complexity of the algorithm, each color plane (red, green, and blue) is confused and diffused together while still remaining efficient in terms of time complexity.

This paper’s presentation will begin by laying the groundwork with an overview of relevant past research in this field. We will then delve into the details of our proposed approach, followed by a presentation of the evaluation results. To solidify the value of our work, we will compare it to existing methods before concluding with a comprehensive discussion and conclusion.

## 2. Background and Current Studies

All encryption methods should be designed to balance complexity and security. This is also true for image encryption. In image encryption, two basic steps are used: confusion and diffusion. In the confusion stage, the positions of the data are changed, but the data themselves are not changed generally. This aims to reduce the high correlation between pixels. In the diffusion stage, mathematical operations are used to ensure that each data bit affects the entire data. This makes the encryption even more secure.

In image encryption, the key plays an important role in achieving the balance between complexity and security. Keys determine the parameters used for the confusion and diffusion operations. Strong keys make it difficult to break the encryption and protect the data from unauthorized access.

The efficient use of each component is an important feature of an elegant image encryption algorithm. An elegant algorithm, in addition to being complex and secure, should also be suitable for practical application. This means that the algorithm should avoid unnecessary steps and offer a reasonable processing time. A literature review was conducted to identify image encryption algorithms based on these principles. These algorithms were evaluated in terms of both complexity and security, as well as practicality. An overview of lightweight encryption algorithms is presented which considers hardware-limited devices, which are the working environment targeted by the proposed study.

In [23], researchers proposed a Josephus navigation-based image encryption approach utilizing a mixed chaotic map. The algorithm consists of a flowchart with a switch statement, three rounds of mixing steps, and one round of spreading steps. It employs four different chaotic maps, with the specific map chosen based on an initial value assignment algorithm proposed in the study. These chaotic maps include the logistic, Chebyshev, sine, and cosine 1D maps. The algorithm first calculates the average of the image pixels, linearizes it, and mixes the resulting linear sequence with Josephus navigation. The parameters of the Josephus tour are calculated using a proposed mathematical model based on the image average, achieving image-specific mixing. The next step involves shuffling rows and columns three times using the four chaotic maps in different ways. Finally, a 2D matrix transformation is performed using two M × N dimensional arrays (where M and N represent the image dimensions) generated with the logistic map. While the algorithm was evaluated using multiple measurement criteria and achieved good security results, it is not suitable for Internet of Things (IoT) systems due to its high time complexity and memory usage. Notably, the proposed algorithm requires iterating through the image at least seven times, significantly impacting the processing speed.

The study in [24] builds upon the shortcomings and improvements identified in [25]. It proposes a dynamic ECB mode which utilizes key-dependent S-boxes for both substitution and generating unique round block keys for each block and iteration. The algorithm requires the input data to be a multiple of 32 bytes; otherwise, padding is necessary. The core operation involves applying a custom round function twice consecutively, followed by block-level permutations.

The results presented in the study are limited and insufficient, hindering direct comparison with other data without reimplementing the algorithm. While the author presents the time complexity as a function of the block size, it is important to note that block ciphers, despite their divide-and-conquer nature, do not inherently have image-independent time complexity. Additionally, the fragmentation process necessitates assembly steps, which involve traversing the image twice. Consequently, considering all operations within blocks and the fragmentation process, the algorithm’s overall time complexity translates to traversing the image at least five times.

In [26], an S-Box image encryption scheme based on the chaotic Duffing oscillator and utilizing the Rijndael transform is proposed. The algorithm begins by rotating the image. Following this, each row is modified using the Duffing equation according to the Rijndael-based S-box transformation. The study evaluates the employed S-Box using criteria such as nonlinearity, bit-independence bit-flip complexity (BIG-SAC), and a strict avalanche criterion. Finally, the image is reconstructed. It is important to note that evaluating the scheme with such a diverse set of criteria necessitates viewing the image at least six times. While the application results suggest that the scheme meets security criteria, the unified average changing intensity (UACI) values fell short of the ideal value, indicating a vulnerability to differential attacks.

The study in [3] proposes a lightweight image encryption algorithm which leverages a logistic map and AES S-box. The algorithm utilizes random sequences generated by the logistic map for both the permutation and substitution steps. Additionally, it employs SHA-256 and incorporates several operations, including two XOR steps, variable key selection, row and column substitutions, and a final chaotic matrix XOR operation. The study thoroughly evaluated the algorithm using various metrics and reported promising results. Notably, the algorithm’s benchmarking times showed favorable performance compared with a 3.5 GHz standard processor. However, considering the individual steps involved (SHA-256, separate row and column permutations, S-Box substitution, and final matrix XOR), the algorithm traverses the encrypted image at least five times, potentially impacting the processing speed.

The study in [27] proposes a lightweight image encryption algorithm specifically designed for biomedical images. The algorithm utilizes a customized 7D hyperchaotic system. Notably, the SHA-512 hash of the image is used to determine the initial values for the chaotic map. The proposed method first extracts features using compressive sensing. Subsequently, the seven outputs generated by the chaotic map are strategically used; the first two are for feature determination, the next three are for column-based permutation and propagation, and the final two are for row-based permutation. Consequently, the algorithm traverses the image at least five times. While the presented results indicated reasonable security, the number of point changes rate (NPCR) and unified average changing intensity (UACI) values exhibited slight deviations from the ideal reference values.

The study in [28] proposes a gray-level image encryption algorithm implemented on a Genesys 2 FPGA, utilizing a combination of a pseudo-random number generator (PRNG), DNA coding, and pixel summation. The Lorenz chaotic system serves as the core for generating randomness. The algorithm comprises 11 stages, with iteration counts ranging from 5 to 11. The process begins with collecting image pixel values. The key is then divided into eight parts and undergoes XOR operations within these segments. Numbers generated by the chaotic system are used for DNA coding. To enhance nonlinearity, a two-stage DNA coding scheme is employed. The maximum number of iterations is 16, determined by the modular 16 operation applied to the sum of the image pixels, which dictates the number of repetitions between stages 5 and 11. These intermediate stages primarily involve generating chaotic numbers and incorporating them as conjugates in the DNA coding process. As this algorithm is implemented in hardware, a direct time complexity comparison with the method proposed in this study is not feasible. While the evaluation metrics presented for the proposed method are limited, it appears to be a successful algorithm overall. However, a potential weakness lies in its key size. Although 2256 bits may be considered sufficient for current security needs, the development of quantum computers poses a threat to key sizes of this level.

The study in [29] proposes a color image encryption algorithm which processes each channel (red, green, blue, and alpha) independently using a dedicated chaotic map. The chosen maps were logistic, sine, Chebyshev, and tent. The study provides a detailed analysis of each map and justifies its specific application within the algorithm. Finally, the Arnold’s cat map transformation is employed during the propagation step. The proposed method was evaluated using a 256 × 256 test image on a processor with a 2.8 GHz clock speed, achieving a promising processing time of 25 ms. Furthermore, the algorithm yielded satisfactory results across all evaluation criteria presented in the study. However, it is worth noting that the method proposed in the present study achieves superior results.

The study in [30] proposes a stereo zigzag algorithm, a pixel-mixing technique frequently employed by researchers for image encryption. This algorithm enables the encryption of multiple images simultaneously. The process begins by arranging four images into a cube-like structure. The Henon map determines the starting point for the stereo zigzag algorithm, which then mixes the image pixels. During the propagation stage, the images are further encrypted using distinct chaotic sequences generated by the Henon map. The algorithm also incorporates the SHA-256 algorithm within its intermediate steps. It is important to note that the algorithm presentation solely utilized grayscale images. While the proposed method has $2^{442}$ key fields, the evaluation results for time complexity fell short of the optimal values, despite being classified as acceptable within the study. The published results (though lacking the processor’s clock frequency) indicate that encrypting a set of four 256 × 256 images takes close to one second. The study denotes the complexity analysis as *O*(k × M × N), where k represents the number of images used in the multi-image encryption process.

A review of the literature revealed several contrasting approaches to chaotic map selection in image encryption algorithms. While some studies considered 1D chaotic maps adequate, others advocated for the use of higher-order alternatives. However, it is crucial to evaluate the core structure (skeleton) and intermediate steps of a proposed encryption scheme independent of the specific chaotic map employed. This approach allows for the independent assessment and improvement of an algorithm’s core logic separate from the specific chaotic map selection. Consequently, the chaotic properties of different maps can be evaluated in dedicated studies.

Therefore, the specific chaotic map used in this study will not be used as a benchmark for the algorithm itself. The primary focus should be on utilizing image encryption evaluation criteria to identify potential shortcomings within the proposed approach.

The field of lightweight image encryption offers a vast array of algorithms. While the literature boasts successful encryption methods, others fall short in terms of performance. This inherent trade-off is often observed in lightweight algorithms, where improvements in one area, such as time complexity, may come at the expense of security. Conversely, algorithms prioritizing security often suffer from high time complexity. Notably, advancements in the pixel mixing stage are scarce, with variations of zigzag [31,32] and spiral [12,33] transformations dominating the landscape. Our previous study [13] further highlighted this trend.

In light of this, the proposed approach strives to achieve a balance between efficiency and time complexity. It presents an elegant solution which boasts low time complexity while meeting security criteria. We believe that this approach can serve as a valuable guide for future research in the field. The algorithm’s performance is evaluated using a comprehensive set of criteria, with the results compared to similar algorithms. The benchmarking results clearly demonstrate the strength of the proposed method.

## 3. Materials and Methods

**Figure 1:**
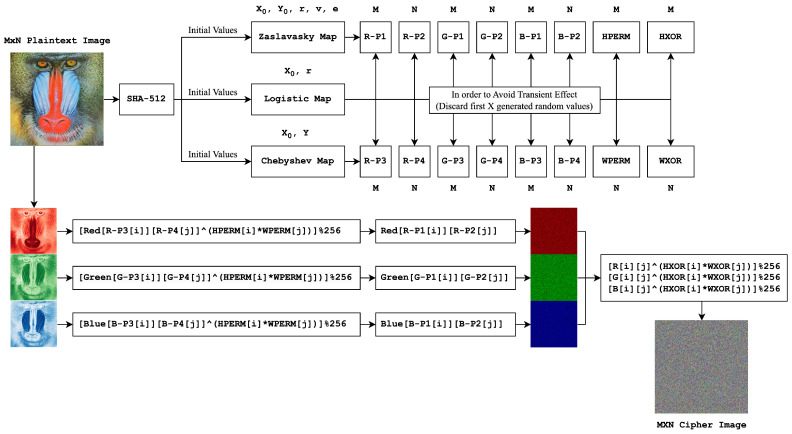
Complete block diagram of the proposed algorithm.

In this study, a color image encryption algorithm with high nonlinearity is proposed, building upon the work in [22]. A visual summary of the proposed algorithm is shown in Figure 1. As can be seen from Figure 1, the approach utilizes three different chaotic maps: a 2D Zaslavsky map, a 1D Chebyshev map, and a 1D logistic map. By employing these maps in various steps and manners, linearity is significantly disrupted. In Figure 1, *M* and *N* represent the sizes of generated random sequences. Using the Zaslavsky map, 5 M-dimensional and 3 N-dimensional random sequences are produced. Similarly, the Chebyshev map generates 3 M-dimensional and 5 N-dimensional random sequences. Finally, the logistic map produces 16 random numbers. These 16 numbers dictate how many generated values will be discarded for the other random number generators, effectively controlling the transient effects of all generated sequences and enhancing security.

The algorithm steps can be summarized as follows:

1. *Initialization:* The SHA512 hash of the encrypted image is used to determine the initial values of the three chaotic maps.
2. *Permutation:* The image is permuted seperately in each color plane using random sequences generated with Zaslavsky and Chebyshev maps. This permutation is performed for each color plane (red, green, and blue) using the Equations (8)–(10).
3. *XOR Operation:* The intermediate image produced in step 2 is subjected to an XOR operation with additional random sequences generated from the chaotic maps, as given in (11), (12), and (13).

### 3.1. Preliminaries

The proposed approach employs three distinct low-complexity chaotic maps. Chaotic maps, which are highly sensitive to initial conditions and control parameters, are unpredictable sequences generated by mathematical formulas. Due to their inherent randomness and a vast array of options available, this study employs a 1D logistic map to prioritize computational efficiency (or the encryption speed, depending on the focus) while maintaining strong security. Their outputs, as shown in Figure 1, are employed in the image’s confusion and diffusion stages together.

The Zaslavsky map is a 2D chaotic map with four parameters, was chosen for its suitable properties. Its equation is presented in (3)–(5).

(3) $$x_{n+1}=x_{n}\cdot v\left(1+\mu \cdot y_{n}\right)+\epsilon \cdot v\cdot \mu \cdot cos\left(2\cdot \pi \cdot x_{n}\right)$$

(4) $$y_{n+1}=e^{r}\cdot \left(y_{n}+\epsilon \cdot \cos\left(2\cdot \pi \cdot x_{n}\right)\right)$$

(5) $$\mu =(1-e^{-r})/r$$

In these equations, ϵ, *v*, μ, and *e* are the control parameters of the map, while $x_{n}$ and $y_{n}$ are the initial values of the map. The nonlinear dynamics and attractors of the adopted Zaslavsky map can be found in [34].

A Chebyshev map is a map with high chaotic properties and a wide chaotic range. However, since it is 1D, it may cause a security vulnerability if not used carefully. The mathematical model of the Chebyshev map is presented in Equation (6):

(6) $$X_{n+1}=\cos\cdot \left(\gamma \cdot \cos^{-1}\left(X_{n}\right)\right)$$

In Equation (6), γ is the control parameter of the map, and $X_{n}$ is the initial value. A Chebyshev map shows chaotic behavior when γ>1. However, when 1<γ<2, the map shows unusual behavior. Therefore, γ>2 should be used for better randomness. The starting point of *X* lies between [−1,1], but it is more chaotic at values close to the extreme points.

Finally, the third map used is a 1D logistic map. The mathematical expression of a 1D logistic map is given in Equation (7):

(7) $$X_{n+1}=\mu \cdot X_{n}\cdot \left(1-X_{n}\right)$$

In (7), the control parameter μ shows chaoticity in the range of 0<μ<4. Here, $x_{n}$ is the initial value, and $x_{n+1}$ is the output value where 0<=n, while $x_{0}$ is in the range of $0<x_{0}<1$.

**Figure 2:**
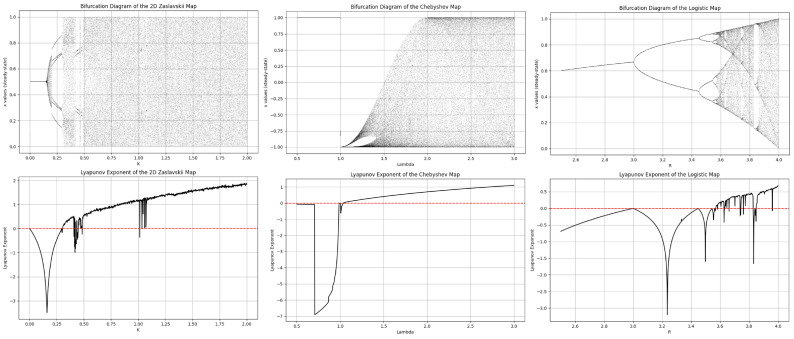
Bifurcation and LE graphics of employed chaotic maps.

Figure 2 illustrates the bifurcation diagrams and Lyapunov exponent (LE) graphs of the three chaotic maps employed in this study. A comparison revealed that the Zaslavsky and Chebyshev maps exhibited a broader chaotic region compared with the logistic map. Furthermore, the logistic map’s Lyapunov exponent remained positive within a more limited range. Consequently, due to its relatively narrower chaotic behavior, the logistic map was exclusively utilized to introduce transient effects and was not incorporated into the permutation or XOR operations.

A random number generator suitable for cybersecurity applications must exhibit strong random properties. Chaotic maps, while classified as pseudo-random number generators, are often favored in resource-constrained environments like the IoT due to their extreme sensitivity to the initial values and ergodic behavior. Table 1 summarizes the NIST 800-22 Rev1a test results for the three chaotic maps employed in this study. Although certain NIST tests recommend a minimum bit length of 1 million, our proposed lightweight image encryption algorithm for IoT systems operates with integers of sizes M and N, which are the dimensions of the input image. Nevertheless, we conducted the NIST tests using 1 million bit sequences generated by the chaotic maps. The results indicate that only the logistic map failed the frequency within a block test, while the other two maps successfully pass all tests.

### 3.2. Proposed Scheme’s Analytical Expression

For improved security, the proposed image encryption algorithm adopts a strategic approach; unique random sequences are employed within each RGB channel, boosting the scheme’s overall nonlinearity. From Equation (8) to Equation (13), we offer a window into the algorithm’s mathematical foundation:

(8) $$A_{r}\left[rp1\left[i\right]\right]\left[rp2\left[j\right]\right]=\left(I_{r}\left[rp3\left[i\right]\right]\left[rp4\left[j\right]\right]⊕\left(hp\left[i\right]\cdot wp\left[j\right]\right)\right)\%256$$

(9) $$A_{g}\left[gp1\left[i\right]\right]\left[gp2\left[j\right]\right]=\left(I_{g}\left[gp3\left[i\right]\right]\left[gp4\left[j\right]\right]⊕\left(hp\left[i\right]\cdot wp\left[j\right]\right)\right)\%256$$

(10) $$A_{b}\left[bp1\left[i\right]\right]\left[bp2\left[j\right]\right]=\left(I_{b}\left[bp3\left[i\right]\right]\left[bp4\left[j\right]\right]⊕\left(hp\left[i\right]\cdot wp\left[j\right]\right)\right)\%256$$

(11) $$C_{red}\left[i\right]\left[j\right]=\left(A_{r}⊕\left(hxor\left[i\right]\cdot wxor\left[j\right]\right)\right)\%256$$

(12) $$C_{greed}\left[i\right]\left[j\right]=\left(A_{g}⊕\left(hxor\left[i\right]\cdot wxor\left[j\right]\right)\right)\%256$$

(13) $$C_{blue}\left[i\right]\left[j\right]=\left(A_{b}⊕\left(hxor\left[i\right]\cdot wxor\left[j\right]\right)\right)\%256$$

In these equations, rp1…4, gp1…4, and bp1…4 represent R−P1…4,G−P1…4, and B−P1…4, respectively, which are random matrices used for permuting color channels, shown in Figure 1. Also, hp and wp are the HPERM and WPERM arrays employed to apply XOR operations to the result of the first step, respectively. Finally, hxor and wxor represent the final XOR operations performed on the resulting image from step 2.

Examining these equations reveals that all six operations can be performed in one cycle. The implementation efficiently achieves an M × N size (image dimensions) by traversing the image once with two nested loops. This results in a simple, applicable, and elegant solution with high nonlinearity.

### 3.3. Demonstration Results

**Figure 3:**
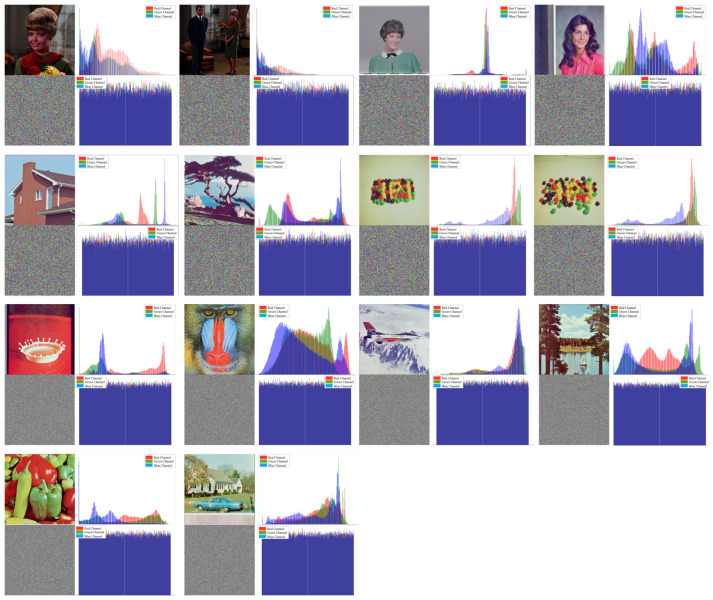
Plaintext and relative cipher image histograms of SIPI dataset images.

**Figure 4:**
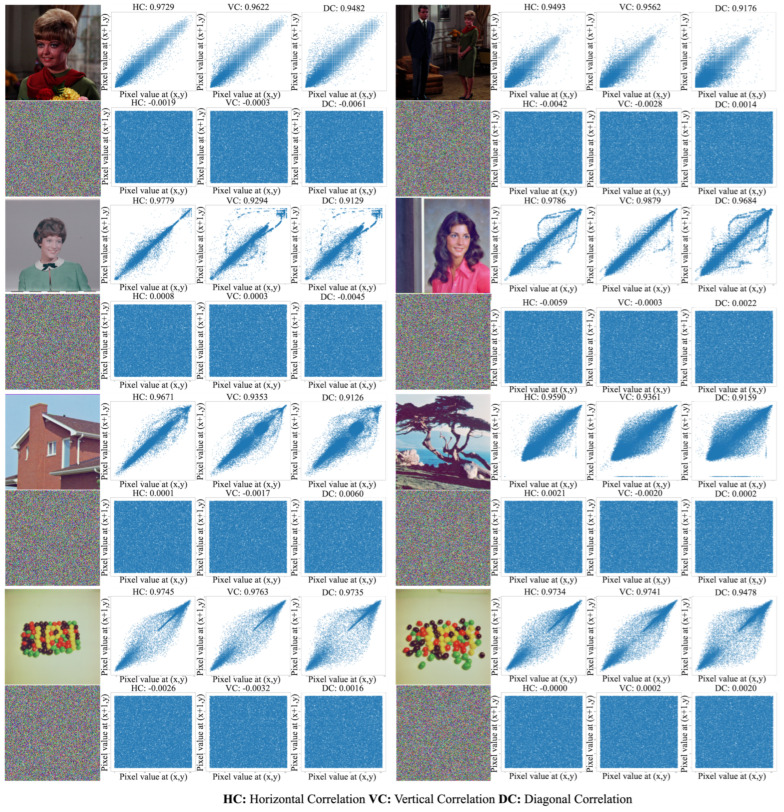
Three phase correlation graphics of SIPI dataset images employed in study.

**Figure 5:**
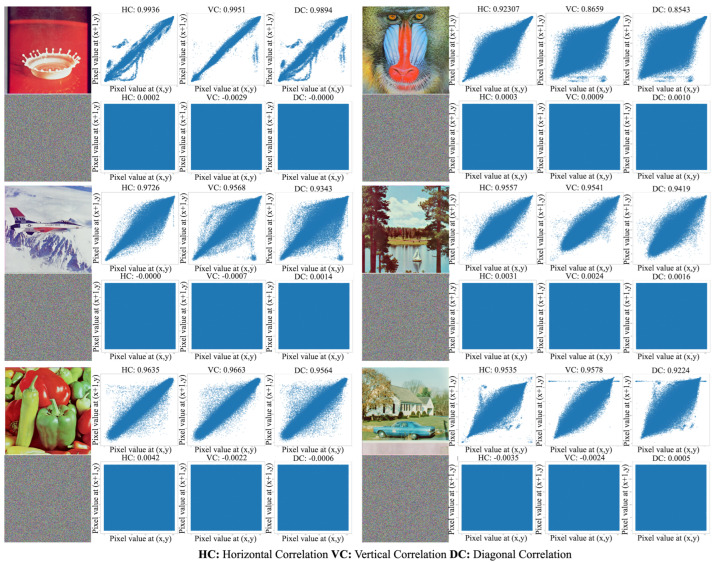
Three phase correlation graphics of SIPI dataset images employed in study continued.

The proposed approach is demonstrated in a lightweight image encryption algorithm. It was implemented using the Java environment. Figure 3, Figure 4 and Figure 5 showcase the encryption and decryption results achieved on test images using the proposed scheme. Notably, all color images in the SIPI Miscellaneous dataset were used in evaluation testing. The figures present the plaintext and encrypted images alongside its corresponding histogram and vertical, horizontal, and diagonal correlation graphs. Correlation graphs in prior research often employed random pixel subsets (1000–5000 pixels). In contrast, our method leverages all image pixels for a more thorough correlation calculation. Despite not showing all correlations, these graphs effectively demonstrate the significant changes in the pixel relationships achieved by the encryption process.

The application results will be presented using several evaluation criteria. These metrics will be primarily examined in the context of statistical and differential analysis.

### 3.4. Histogram Analysis

A histogram of a digital image represents the distribution of pixel values. For visual images, the distribution of pixel values follows a certain discernable pattern. Statistical attack is a common method for finding statistical clues to break a cryptosystem. A secure encryption system should ensure that the encrypted image has a uniform frequency distribution and provides as little statistical information as possible. The second column of the application result images presented in Figure 3 shows the histogram results of the plaintext and encrypted images. As can be seen in this figure, the proposed algorithm produced similar graphs for all encrypted images, regardless of how different the histogram of the plaintext image to be encrypted was. Therefore, it can be concluded that the proposed method is successful in histogram analysis.

### 3.5. Correlation Coefficient Analysis

Due to the inherent nature of images, adjacent pixels exhibit high correlation. The correlation coefficient is a numerical measure used to quantify the statistical relationship between two variables. High correlation implies that attackers can attempt to predict adjacent pixel values using probability theory. A perfect image encryption algorithm should reduce the correlation between adjacent pixels and provide a minimal correlation coefficient. The correlation values between the pixels of an image are given in (14)–(16).

(14) $$\rho_{xy}=\frac{E\left[\left(x-E\left(x\right)\left(y-E\left(y\right)\right)\right)\right]}{\sqrt{D\left(x\right)\left(y\right)}}$$

(15) $$\mathrm{where}\;E\left(x\right)=\frac{1}{N}\sum_{1}^{N}x_{i}$$

(16) $$\mathrm{and}\;D\left(x\right)=\frac{1}{N}\sum_{1}^{N}{\left[\left(x_{i}-E\left(x_{i}\right)\right)\right]}^{2}$$

Unlike typical applications which calculate correlation coefficients with a subset of randomly chosen pixels (e.g., 3000 or 5000), this approach analyzes all pixel values to capture the image’s complete correlation characteristics. Figure 4 and Figure 5 present the horizontal, vertical, and diagonal correlation graphs of the plaintext and cipher images in the third column. Additionally, Table 2 provides the numerical correlation analysis results. Table 2 presents the correlation coefficients for all color images in the SIPI dataset. To enhance clarity, the average correlation values for the 14 images are provided in the last row. By examining these results, the effectiveness of the proposed scheme against statistical attacks becomes more evident.

### 3.6. Information Entropy Analysis

In image encryption, entropy, a measure of randomness, plays a vital role. It helps conceal information from unauthorized access. By aiming to increase an image’s entropy through the encryption process, the algorithm essentially transforms an image into a more unpredictable state, making it significantly harder for attackers to decipher its contents. The maximum entropy value of a dataset depends on the number of unique events (or classes) it contains. Entropy, a measure of uncertainty or randomness in information theory, is calculated using Equation (17).

(17) $$H\left(K\right)=\sum_{i=0}^{n}P\left(K_{i}\right)log_{b}\frac{1}{P\left(K_{i}\right)}$$

where $p(K_{i})$ is the probability of event (or class) $K_{i}$ and *n* is the total number of events. The logarithm base (*b*) typically takes values of 2 (entropy in bits), e (entropy in nats), or 10 (entropy in dits). Entropy reaches its maximum value when all events are equally likely ( i.e., $p(K_{i})=1/n$ for each $K_{i}$). This scenario represents the most uncertain state in the dataset. In summary, the maximum entropy of a dataset varies depending on the number of unique events and the chosen logarithm base.

Except for the alpha channel (which controls transparency), a pixel typically consists of 24 bits, with 8 bits for each color channel (red, green, and blue). Therefore, the maximum entropy value of an image would be eight. Table 3 presents the entropy values of the proposed algorithm for both plaintext and cipher images across the entire SIPI dataset’s color images. The table also includes a column showing the deviation of these values from the maximum entropy. As can be seen in Table 3, the average entropy value for color images in the SIPI dataset was 7.9993, with an average deviation of 0.0007 from the maximum value.

The mean squared error (MSE) measures the average squared difference between the pixel values of two images. A higher MSE indicates a larger difference between the images. However, for interpreting image quality, the peak signal-to-noise ratio (PSNR) is often used instead of solely relying on the MSE value. The PSNR incorporates the maximum possible pixel value, making it easier to compare images with different bit depths. The MSE and PSNR formulae are given in Equations (18) and (19), respectively. The PSNR values of the proposed encryption scheme are presented in Table 3:

(18) $$\mathrm{MSE}=\frac{1}{M\cdot N}\sum_{j=1}^{N}\left|I_{0}\left(i,j\right)-I_{E}\left(i,j\right)\right|$$

(19) $$\mathrm{PSNR}=10\cdot log_{10}\left(\frac{255^{2}}{\mathrm{PSNR}}\right)$$

### 3.7. Differential Analysis

Plaintext sensitivity is an indicator of an image encryption system’s resistance to differential attacks. It is measured by quantifying the difference between images generated by encrypting two plain images which differ by only one bit [4]. Two common metrics used to measure this difference are the number of pixel change rate (NPCR) and unified average changing intensity (UACI). These metrics are defined mathematically in Equations (20) and (21):

(20) $$\mathrm{UACI}=\frac{1}{N}\sum_{i,j}\frac{|D_{1}\left(i,j\right)-D_{2}\left(i,j\right)|}{256}*100\%$$

(21) $$\mathrm{NPCR}=\frac{\sum_{i,j}D\left(i,j\right)}{W*H}*100$$

(22) $$\mathrm{where}\;D\left(i,j\right)=\left\{\begin{array}{c} 0,C_{1}\left(i,j\right)=C_{2}\left(i,j\right)\\ 1,\mathrm{otherwise} \end{array}\right.$$

The UACI measures the average difference in intensity between corresponding pixels of two images. It indicates how much a single pixel change in the original image affects the encrypted image. Conversely, the NPCR reflects the percentage of pixel locations where the encrypted image changes after a single bit flip in the original image. It serves as a measure of the algorithm’s resistance to differential attacks, where attackers attempt to recover information by manipulating the original image slightly. The NPCR and UACI values of the proposed scheme are presented in Table 3 as NPCR Pixel and UACI Pixel, respectively. The other NPCR Key and UACI Key values are the key sensitivity analysis results of the proposed scheme.

Building upon the findings in [35], which established ideal PCNR and UACI values at 99.6094070% and 33.4635070%, respectively, Table 3 demonstrates that the proposed framework achieved performance metrics close to these reference values. This proximity suggests strong encryption capabilities. The average NPCR and UACI values deviated from the expected values by 0.0028 and 0.0055, respectively.

### 3.8. Key Sensitivity Analysis

A critical metric for key sensitivity analysis is the extent to which a minor change in the key impacts the encryption process’s output. To evaluate the key sensitivity of the proposed approach, we employed NPCR and UACI tests. As presented in Table 3, the NPCR Key and UACI Key values were calculated using a key change at a level of $10^{-15}$.

A good image encryption system requires high key sensitivity to ensure thorough mixing of the key with the plaintext. Our scheme utilizes nine keys. Key sensitivity is measured by the effect a minute change in any key field has on the encrypted image. Here, we calculated the NPCR and UACI values between the cipher image generated with a $10^{-15}$ change in the $x_{0}$ parameter of the Zaslavsky map and the original encrypted image (presented in Table 3 as NPCR Key and UACI Key, respectively). The results demonstrate the proposed system’s strong key sensitivity and comprehensive attack resistance. The average NPCR and UACI values deviated from the expected values by 0.0021 and 0.0068, respectively.

### 3.9. Key Space Analysis

In image encryption, the key space size is typically defined by the total number of bits used in the key. This scheme utilizes a two-part key: the initial values of the different chaotic maps and the SHA-512 hash value of the plaintext image. Since the initial values had double precision, their space was $2^{288}$. The space of the 512 bit SHA-512 hash value was $2^{5}12$. Therefore, the entire scheme’s key space became $2^{800}$. With a key space of $2^{800}$, the proposed scheme significantly surpassed the minimum threshold of $2^{256}$ [14,28,36], which is considered resistant to brute-force attacks. This vast key space effectively renders brute-force attacks computationally infeasible, ensuring a high level of security against such attempts. For post-quantum security, key space also plays a vital role.

### 3.10. Robustness Analysis

**Figure 6:**
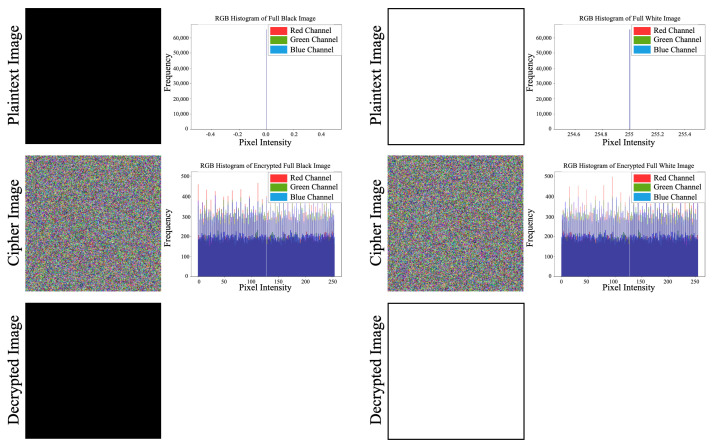
Full black and white image encryption, decryption and histogram results.

The robustness of a secure image encryption algorithm is often evaluated using its performance on extreme cases. Here, we analyzed the encryption and decryption performance for black-and-white images. Figure 6 presents the results. It shows the encryption and decryption of specially designed 256 × 256 black-and-white images, along with their corresponding histogram plots. As can be seen in the figure, the proposed method successfully handled these types of images.

**Figure 7:**
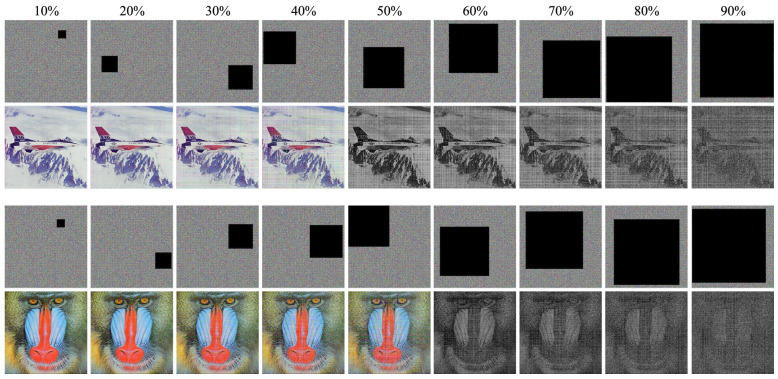
Decryption result for different percentage data loss on cipher image.

**Figure 8:**
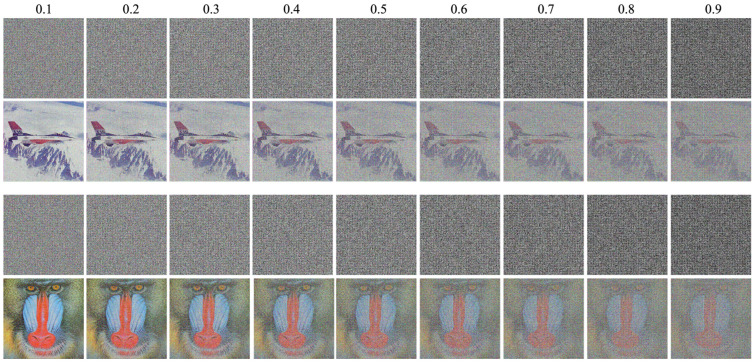
Decryption result for different percentage Salt and Peppers noise on cipher image.

The proposed algorithm’s robustness was further evaluated by its ability to decrypt chip images with data loss and noise. Two tests were conducted. In the first, cipher images with random data loss ranging from 10% to 90% were decrypted (results in Figure 7). The second test involved encrypted images infused with varying degrees of salt-and-pepper noise (0.1–0.9). The decrypted images are shown in Figure 8. In both tests, the proposed approach achieved remarkable resilience. As evident from the figures, even with 90% data loss or 0.9 salt-and-pepper noise, the original image’s silhouette remained discernible. To ensure generalizability, these tests utilized the two most frequently used images (baboon and airplane) from the test set. These successful results demonstrate that the proposed algorithm is not only elegant, efficient, and secure but also remarkably robust against data loss and noise.

### 3.11. Efficiency Analysis

The proposed image encryption scheme demonstrates exceptional efficiency in terms of time complexity. All operations can be executed concurrently, minimizing the processing time. Tests on a 2.4 GHz processor revealed an average encryption time of 29 ms and an average decryption time of 25 ms for 100 runs. While these specific results were excluded from formal comparisons for objectivity, the method boasted a favorable time complexity of *O*(M × N), signifying its efficient operation.

Table 4 compares the time complexity and execution times of the proposed approach with similar studies. While direct comparisons of execution times across different hardware environments can be challenging, we attempted to mitigate this by considering the processor GHz frequencies of the systems used. Additionally, the table presents the time complexity analyses of the algorithms based on the image size, providing a more comprehensive evaluation.

The comparative analysis was conducted using 512 × 512 test images, as this was the common image size for the studies being evaluated. However, to assess the algorithm’s performance on different image sizes, we also tested it on 256 × 256 and 1024 × 1024 images. The results demonstrated execution times of 21 ms and 32 ms, respectively, on our test system.

## 4. Comparison

Due to the inherent variability in image encryption algorithms arising from differences in approach, application area, and test images, image-by-image comparisons can be challenging. To address this, Table 5 presents a comparison based on the average values of the evaluation criteria presented in this work. The table includes results from state-of-the-art image encryption algorithms alongside the evaluation metrics of the method proposed in this study. To ensure a fair comparison in this study, the evaluations of other image encryption algorithms were based on metrics calculated using the same methods employed in this work. This approach ensured consistency across comparisons. The averages were calculated while considering the number of test images used in each compared study. For instance, correlation values were summed as absolute values, and the average result was obtained for each study.

A comparison of our proposed encryption framework with existing lightweight image encryption methods revealed it as a viable alternative, even when the image was processed only once. While the chaotic maps employed in this work were relatively low-dimensional, increasing the number of intermediate layers or utilizing higher-order chaotic maps could undoubtedly enhance the security of our approach to an even greater extent.

As shown in Table 5, the metric results of our proposed approach closely aligned with those of other recent state-of-the-art methods. Furthermore, when considering the single image pass and superior performance in two key metrics, the efficiency of our framework becomes evident. Importantly, the $2^{800}$ key fields achieved using simple chaotic maps can be significantly expanded by incorporating higher-order chaotic maps, potentially enhancing the algorithm’s resistance to post-quantum attacks.

## 5. Discussion

This study proposes a foundation for lightweight image encryption algorithms which leverages three different chaotic maps. By utilizing these maps strategically, the scheme enhances security while maintaining simplicity of application and efficiency. In its most basic form, the encryption scheme uses 16 arrays: 8 for the image width and 8 for the height. This might raise concerns about memory usage. However, the system’s memory footprint in this configuration is approximately 16 MB, which is demonstrably low. Consequently, the proposed approach demonstrates efficiency in both time and space complexity.

The proposed algorithm is specifically designed for two-dimensional image data. While theoretically applicable to video data (essentially a sequence of images), two primary challenges impede its direct use—compressed video formats and high throughput—as well as the need for temporal consistency. Videos are typically stored in compressed formats (e.g., MPEG and H.264) and involve high data rates, requiring the algorithm to be compatible with compression standards and maintain efficiency for large datasets. Moreover, the temporal relationships between video frames necessitate specialized encryption techniques beyond individual frame encryption.

Audio data, being a one-dimensional time series, would require substantial transformations before adapting the image encryption algorithm. This added complexity renders it unsuitable for resource-constrained IoT systems prioritizing simplicity and low cost. Specialized algorithms designed for voice data would be more appropriate in such environments.

Given the specialized nature of IoT sensor networks typically focused on a single task, the proposed algorithm is well suited for securing image data during transmission and storage within these networks.

One potential criticism could be the use of low-dimensional chaotic maps. While these offer advantages in terms of key space and system complexity compared with high-dimensional ones [4], they are particularly suitable for the resource-constrained devices targeted in this work (e.g., IoT). More importantly, the proposed method employs nonlinear matrix transitions, effectively eliminating any linearity within the system. Additionally, it addresses the concern of limited key space in low-dimensional maps. The resulting key space of $2^{800}$ is more than sufficient for IoT systems. It is important to remember that this study presents a foundational framework. The system complexity and key space can be further increased using higher-dimensional chaotic maps depending on specific security requirements.

A common question is why correlation coefficient graphs are analyzed using all pixels instead of a randomly selected subset. We opted to include all pixels for two primary reasons. First, we believe that pixel selection using any pseudo-random number generator might introduce bias. Therefore, a comprehensive analysis using all pixels provides a more equitable assessment. Second, while correlation graphs offer a visual representation, the numerical data themselves are the crux of the comparison. Thus, the correlation values obtained for the proposed method are presented in Table 2 for a more quantitative evaluation.

## 6. Conclusions

The growing prevalence of Internet of Things (IoT) devices intensifies the need for secure yet lightweight encryption algorithms. These algorithms must balance strong security with the limited hardware resources of IoT devices. In this context, chaos theory offers a promising solution.

This study proposed a lightweight image encryption framework which leverages different chaotic maps. The framework exhibits exceptional efficiency, as demonstrated by various evaluation criteria, achieving high values in a single round.

Furthermore, the framework offers flexibility by allowing for increased complexity through higher-dimensional chaotic maps or additional iterations, catering to specific security needs. Notably, the proposed framework prioritizes not only efficiency and security but also elegance in its design and application, making it a valuable solution for IoT image encryption. The proposed framework excels in terms of efficiency, boasting a time complexity of O(M×N) and a space complexity of O(M+N), making it ideal for resource-constrained environments. Additionally, it achieved near-perfect entropy, with a difference of only 0.0007 from the maximum value. Furthermore, the results surpassed expectations for both the NPCR and UACI metrics, exhibiting gaps of only 0.0021 and 0.0068 from their ideal values, respectively. These exceptional results solidify the proposed framework’s effectiveness for lightweight image encryption.

## Figures and Tables

**Table 1:** NIST SP 800-22 rev1a test result of employed chaotic maps.

Test Name	Zaslavsky Map	Chebyshev Map	Logistic Map
Frequency	Pass	Pass	Pass
Frequency within a Block	Pass	Pass	Failed
Runs	Pass	Pass	Pass
Longest Run of Ones	Pass	Pass	Pass
Binary Matrix Rank	Pass	Pass	Pass
Discrete Fourier	Pass	Pass	Pass
Non-Overlapping Template Matching	Pass	Pass	Pass
Overlapping Template Matching	Pass	Pass	Pass
Universal	Pass	Pass	Pass
Linear Complexity	Pass	Pass	Pass
Serial	Pass	Pass	Pass
Approximate Entropy	Pass	Pass	Pass
Cumulative Sums	Pass	Pass	Pass
Random Excursions	Pass	Pass	Pass
Random Excursions Variant	Pass	Pass	Pass

**Table 2:** Correlation coefficient results of proposed algorithm.

Image	Hor. Corr.		Ver. Corr.		Dia. Corr.	
	Plaintext	Cipher	Plaintext	Cipher	Plaintext	Cipher
4.1.01	0.9629	−0.0019	0.9622	−0.0003	0.9482	−0.0061
4.1.02	0.9493	−0.0042	0.9562	−0.0028	0.9176	0.0014
4.1.03	0.9779	0.0008	0.9294	0.0003	0.9129	−0.0045
4.1.04	0.9786	−0.0059	0.9879	−0.0003	0.9684	0.0022
4.1.05	0.9671	0.0001	0.9353	−0.0017	0.9126	0.0060
4.1.06	0.9590	0.0021	0.9361	−0.0020	0.9159	0.0002
4.1.07	0.9745	−0.0026	0.9763	−0.0032	0.9537	0.0016
4.1.08	0.9734	−0.0000	0.9741	0.0002	0.9478	0.0020
4.2.01	0.9936	0.0002	0.9951	−0.0029	0.9894	−0.0000
4.2.03	0.9231	0.0003	0.8660	0.0009	0.8543	0.0010
4.2.05	0.9726	−0.0000	0.9568	−0.0007	0.9343	0.0015
4.2.06	0.9558	0.0032	0.9541	0.0024	0.9420	0.0016
4.2.07	0.9635	0.0041	0.9663	−0.0021	0.9564	−0.0006
house	0.9536	−0.0035	0.9579	−0.0024	0.9224	0.0005
Average	0.9646	−0.0005	0.9538	−0.001	0.9339	0.0004

**Table 3:** Evaluation results of the proposed approach.

SIPI Image	Plaintext Entropy	Cipher Entropy	Entropy Deviation	PSNR	NPCR Pixel	UACI Pixel	NPCR Key	UACI Key
4.1.01	6.8981	7.9984	0.0016	7.2897	99.5895	33.4347	99.6220	33.3791
4.1.02	6.2945	7.9991	0.0009	6.2518	99.6098	33.5497	99.5981	33.4934
4.1.03	5.9709	7.9990	0.0010	9.9564	99.6185	33.5128	99.6058	33.4923
4.1.04	7.4269	7.9990	0.0010	8.8215	99.6089	33.4365	99.5819	33.4341
4.1.05	7.0686	7.9990	0.0010	8.9295	99.5967	33.4517	99.6089	33.5056
4.1.06	7.5371	7.9989	0.0011	8.1627	99.6144	33.3184	99.5966	33.3930
4.1.07	6.5835	7.9991	0.0009	8.6094	99.6205	33.4366	99.6114	33.4796
4.1.08	6.8527	7.9991	0.0009	8.6448	99.6170	33.4806	99.6124	33.4764
4.2.01	7.2428	7.9997	0.0003	7.6329	99.6206	33.4769	99.6049	33.4162
4.2.03	7.7624	7.9998	0.0002	8.7700	99.6288	33.4559	99.6215	33.4799
4.2.05	6.6639	7.9998	0.0002	7.9830	99.6081	33.4997	99.6117	33.4637
4.2.06	7.7622	7.9998	0.0002	8.0845	99.6135	33.5064	99.6173	33.5072
4.2.07	7.6698	7.9997	0.0003	8.0827	99.6236	33.5133	99.6055	33.5188
house	7.4858	7.9998	0.0002	8.4707	99.6009	33.4934	99.6044	33.4326
Average	7.0871	7.9993	0.0007	8.2635	99.6122	33.4690	99.6073	33.4622

**Table 4:** Efficiency comparison of proposed scheme with similar studies.

Study	Time Complexity	Execution Time
Study [3]	8 × M × N	42 ms
Study [12]	12 × M × N	583 ms
Study [37]	7 × M × N	666 ms
Study [38]	8 × M × N	34 ms
Study [39]	12 × M × N	1160 ms
Proposed	M × N	25

**Table 5:** Comparison of the proposed method with image encryption algorithms in the literature.

Evaluation Metric	Ref. [3]	Ref. [14]	Ref. [29]	Ref. [40]	Ref. [41]	Ref. [42]	Ref. [43]	Ref. [44]	Proposed
Correlation	0.3645	0.0009	0.0069	0.0047	0.0036	0.004	0.0046	0.0018	0.00063
PSNR	9.2098	8.6267	6.569	-	-	-	-	-	8.2635
Entropy	7.9987	7.9983	7.9987	7.9964	7.9985	7.9025	7.9981	7.9971	7.9993
NPCR Pixel	99.6153	99.5211	99.652	-	99.6089	99.7272	98.3051	99.6143	99.6122
UACI Pixel	33.4718	33.1778	33.5244	-	33.4658	33.473	32.4134	33.5002	33.4690
NPCR Key	-	-	-	-	-	-	-	-	99.6073
UACI Key	-	-	-	-	-	-	-	-	33.4622
Key Space	$2^{256}$	$2^{1116}$	-	$2^{600}$	$2^{471}$	-	$2^{384}$	-	$2^{800}$

## Data Availability

The data presented in this study are available at https://sipi.usc.edu/database/database.php?volume=misc (accessed on 11 September 2024).
